# Hexa­kis­(1*H*-imidazole-κ*N* ^3^)iron(II) sulfate–1*H*-imidazole (1/2)

**DOI:** 10.1107/S1600536811043169

**Published:** 2011-10-29

**Authors:** Alexandra Nistor, Sergiu Shova, Maria Cazacu, Alina Lazar

**Affiliations:** a Institute of Macromolecular Chemistry ‘Petru Poni’, Inorganic Polymers Department, 41A Grigore Ghica Voda Alley, Iasi-700487, Romania; b Institute of Applied Physics of the Academy of Science of Moldova, 5 Academiei Street, Chisinau MD-2028, Republic of Moldova

## Abstract

The asymmetric unit of the title compound, [Fe(C_3_H_4_N_2_)_6_]SO_4_·2C_3_H_4_N_2_, contains two complex cations, two sulfate anions and four imidazole mol­ecules. In both cations, the Fe^II^ atom is coordinated by six monodentate imidazole ligands and exhibits a slightly distorted octa­hedral coordination geometry. The Fe—N distances [2.184 (4)–2.218 (4) Å] point to a high-spin state of the Fe^2+^ ions. N—H⋯O hydrogen bonds between the ionic components generate a three-dimensional framework containing corrugated channels along [001], which are filled by **N—H⋯N** hydrogen-bonded imidazole chains.

## Related literature

For the crystal structures of other hexa­kis­(imidazole)­iron(II) salts, see: Carver *et al.* (2003 ▶); Jian *et al.* (2004 ▶). For spin crossover in complexes with the FeN_6_ core, see: Gütlich & Goodwin (2004 ▶); Lemercier *et al.* (2006 ▶). For the influence of counter-ions and solvent mol­ecules on spin crosover behaviour, see: Bousseksou *et al.* (1996 ▶).
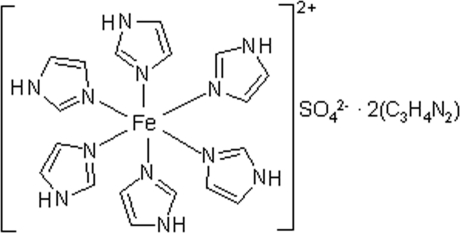


## Experimental

### 

#### Crystal data

[Fe(C_3_H_4_N_2_)_6_]SO_4_·2C_3_H_4_N_2_
*M* *_r_* = 696.57
Triclinic, 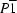

*a* = 15.4091 (8) Å
*b* = 15.4436 (7) Å
*c* = 15.9883 (11) Å
α = 69.813 (5)°
β = 69.949 (5)°
γ = 73.214 (4)°
*V* = 3291.3 (3) Å^3^
*Z* = 4
Mo *K*α radiation
μ = 0.58 mm^−1^
*T* = 200 K
0.20 × 0.15 × 0.15 mm

#### Data collection

Oxford Diffraction Xcalibur E diffractometer
Absorption correction: multi-scan (*CrysAlis PRO*; Oxford Diffraction, 2009 ▶) *T*_min_ = 0.901, *T*_max_ = 0.917
15987 measured reflections
10955 independent reflections
5185 reflections with *I* > 2σ(*I*)
*R*_int_ = 0.046

#### Refinement

*R*[*F* ^2^ > 2σ(*F* ^2^)] = 0.065
*wR*(*F* ^2^) = 0.107
*S* = 0.85
10955 reflections
829 parameters
H-atom parameters constrained
Δρ_max_ = 0.63 e Å^−3^
Δρ_min_ = −0.50 e Å^−3^

### 

Data collection: *CrysAlis PRO* (Oxford Diffraction, 2009 ▶); cell refinement: *CrysAlis PRO*; data reduction: *CrysAlis PRO*; program(s) used to solve structure: *SHELXS97* (Sheldrick, 2008 ▶); program(s) used to refine structure: *SHELXL97* (Sheldrick, 2008 ▶); molecular graphics: *ORTEP-3* (Farrugia, 1997 ▶); software used to prepare material for publication: *SHELXL97*.

## Supplementary Material

Crystal structure: contains datablock(s) I, global. DOI: 10.1107/S1600536811043169/gk2405sup1.cif

Structure factors: contains datablock(s) I. DOI: 10.1107/S1600536811043169/gk2405Isup3.hkl

Additional supplementary materials:  crystallographic information; 3D view; checkCIF report

## Figures and Tables

**Table 1:** Hydrogen-bond geometry (Å, °)

*D*—H⋯*A*	*D*—H	H⋯*A*	*D*⋯*A*	*D*—H⋯*A*
N2—H49⋯O4	0.88	1.84	2.679 (5)	160
N4—H50⋯O5	0.88	1.89	2.762 (5)	170
N6—H51⋯O3^i^	0.88	2.09	2.949 (7)	163
N8—H52⋯O1^ii^	0.88	1.94	2.822 (5)	176
N10—H53⋯O7^iii^	0.88	1.85	2.711 (6)	166
N12—H54⋯O7^iv^	0.88	2.10	2.867 (7)	145
N12—H54⋯O5^iv^	0.88	2.61	3.434 (7)	157
N14—H55⋯O8^v^	0.88	1.85	2.716 (5)	167
N16—H56⋯O1^vi^	0.88	2.22	3.074 (6)	163
N16—H56⋯O4^vi^	0.88	2.26	2.942 (6)	135
N18—H57⋯O6^vii^	0.88	2.07	2.937 (6)	169
N18—H57⋯O8^vii^	0.88	2.47	3.100 (6)	129
N20—H58⋯O6	0.88	2.21	3.071 (6)	166
N20—H58⋯O7	0.88	2.45	3.120 (6)	133
N22—H59⋯O2	0.88	2.18	2.939 (6)	144
N22—H59⋯O3	0.88	2.37	3.186 (6)	155
N24—H60⋯O2^viii^	0.88	1.84	2.710 (6)	168
N26—H61⋯N29	0.88	1.95	2.825 (6)	177
N27—H62⋯N25	0.88	2.04	2.875 (6)	158
N30—H63⋯N32^ix^	0.88	2.02	2.869 (6)	160
N31—H64⋯N28	0.88	1.92	2.800 (6)	174

Symmetry codes: (i) 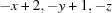; (ii) 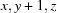; (iii) 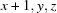; (iv) 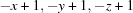; (v) 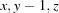; (vi) 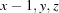; (vii) 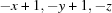; (viii) 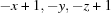; (ix) 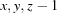.

**Fig. 1.**
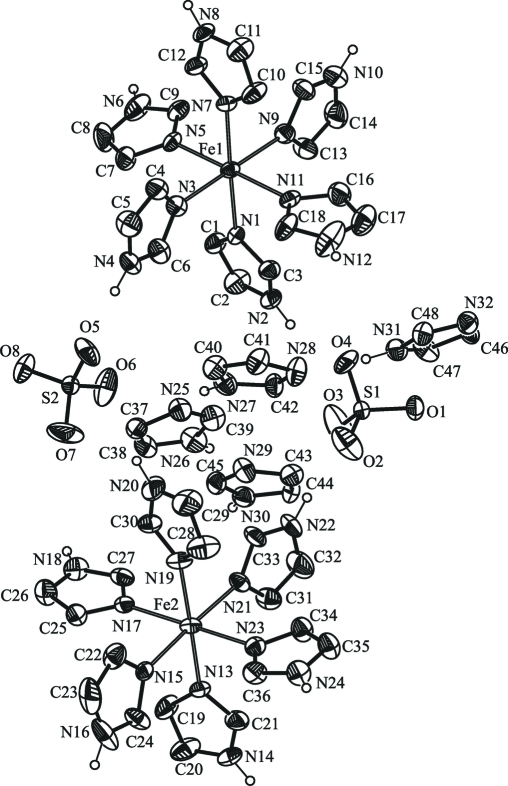
The structure of the asymmetric unit for [Fe(C3H4N2)6]SO4.2C3H4N2 with displacement ellipsoids shown at the 50% probability level.

**Fig. 2.**
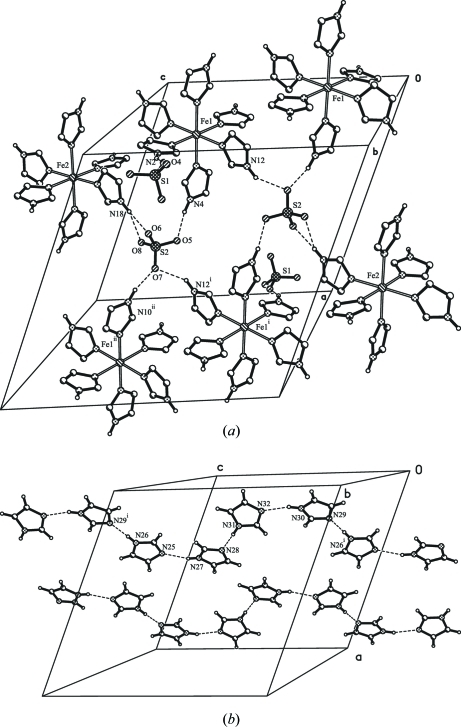
Crystal packing diagrams showing three-dimensional framework formed by hydrogen-bonded cations and anions (a) and the [0 0 1] chain of hydrogen- bonded imidazole molecules (b). Only H atoms involved in hydrogen bonding are shown. Hydrogen bonds are shown with dashed lines. Symmetry code: (i)1 - x, 1 - y, 1 - z; (ii) x-1, y, z.

## supplementary crystallographic information

### Comment

As part of our research of iron(II) complexes with mono- and polydentate N-containing ligands we report the synthesis and crystal structure of [Fe(Im)_6_]SO_4_^.^2Im, where Im = imidazole. The cationic complexes [Fe(Im)_6_]^2+^ characterized by an [FeN_6_] coordination core are increasingly investigated as spin crossover (SC) materials, because their bistability (LS, S=0, ^1^A_1_ <-> HS, S=2, ^5^T_2 g_) is easily triggered thermally, magnetically, by pressure or by light irradiation (Gütlich & Goodwin, 2004; Lemercier *et al.*, 2006).

The presence of sulfate counteranion in the title compound with respect to the earlier studied hexakis(imidazole)iron(II) dinitrate, [Fe(Im)_6_]2(NO_3_) (Carver *et al.*, 2003) and hexakis(imidazole)iron(II) dichloride tetrahydate,[Fe(Im)_6_]Cl_2_ 4H_2_O (Jian *et al.*, 2004) is useful as the cationic species could exhibit a large variety of SC behaviors, depending on the non-coordinated counter anions and solvate molecules (Bousseksou *et al.*, 1996). The asymmetric unit of the title compound is depicted in Fig. 1. The Fe^II^ atoms have a slightly distorted octahedral environment, being each coordinated by six monodentate imidazole ligands. The interatomic distances of the FeN_6_ cores allow to conclude that the Fe^2+^ cation is in a high-spin state. Indeed, the average Fe—N bond lenghs for the two [Fe(Im)_6_]^2+^ units A and B is equal to 2.200 (4) Å and 2.210 (4) Å, respectively. The ionic components in the crystal form a three dimensional framework *via* N—H···O hydrogen bonds (Fig. 2a) and the imidazole molecules form hydrogen bonded chains running in the channels of ionic framework (Fig. 2 b). The geometry of hydrogen bonds is listed in Table 1.

### Experimental

Colourless and transparent single crystals of the title compound were obtained as a principal product from the reaction of iron(III) sulfate pentahydrate (0.06 mmol/0.024 g) and imidazole (0.36 mmol/0.0245 g) in the presence of hexamethyldisilazane (0.06 mmol/0.0096 g), terephtalic acid (0.06 mmol/0.00996 g) and dimethylformamide (6 ml) as solvent. The reaction mixture was placed in a glass reactor, which was sealed and kept at 354 K for 24 h. Then, the mixture was cooled to room temperature with a cooling rate of 0.1 K/min and maintained in these conditions for 20 days. Suitable crystals for *X*– ray analysis were separated at the bottom of the flask. Elemental analysis calculated for (C_24_H_32_N_16_FeSO_4_): C 41.39%, H, 4.63%, N, 32.17%, found: C 41.50%; 4.60%, N 32.11%.

### Refinement

The H atoms were positioned geometrically and refined using a riding model approximation with C—H = 0.95 Å, N—H = 0.88 Å and with *U*_iso_(H) = 1.2 *x U*_eq_(C,*N*).

### Crystal data

**Table:**

[Fe(C_3_H_4_N_2_)_6_]SO_4_·2C_3_H_4_N_2_	*Z* = 4
*M**_r_* = 696.57	*F*(000) = 1448
Triclinic, *P*1	*D*_x_ = 1.406 Mg m^−^^3^
*a* = 15.4091 (8) Å	Mo *K*α radiation, λ = 0.71073 Å
*b* = 15.4436 (7) Å	Cell parameters from 3265 reflections
*c* = 15.9883 (11) Å	θ = 2.9–29.1°
α = 69.813 (5)°	µ = 0.58 mm^−^^1^
β = 69.949 (5)°	*T* = 200 K
γ = 73.214 (4)°	Prism, colourless
*V* = 3291.3 (3) Å^3^	0.20 × 0.15 × 0.15 mm

### Data collection

**Table:**

Oxford Diffraction Xcalibur E diffractometer	10955 independent reflections
Radiation source: fine-focus sealed tube	5185 reflections with *I* > 2σ(*I*)
graphite	*R*_int_ = 0.046
Detector resolution: 16.1593 pixels mm^-1^	θ_max_ = 25.0°, θ_min_ = 2.9°
ω scans	*h* = −14→18
Absorption correction: multi-scan (*CrysAlis PRO*; Oxford Diffraction, 2009)	*k* = −18→18
*T*_min_ = 0.901, *T*_max_ = 0.917	*l* = −19→18
15987 measured reflections	

### Refinement

**Table:**

Refinement on *F*^2^	Primary atom site location: structure-invariant direct methods
Least-squares matrix: full	Secondary atom site location: difference Fourier map
*R*[*F*^2^ > 2σ(*F*^2^)] = 0.065	Hydrogen site location: inferred from neighbouring sites
*wR*(*F*^2^) = 0.107	H-atom parameters constrained
*S* = 0.85	*w* = 1/[σ^2^(*F*_o_^2^) + (0.0001*P*)^2^] where *P* = (*F*_o_^2^ + 2*F*_c_^2^)/3
10955 reflections	(Δ/σ)_max_ = 0.001
829 parameters	Δρ_max_ = 0.63 e Å^−^^3^
0 restraints	Δρ_min_ = −0.50 e Å^−^^3^

### Special details

**Table:**

Geometry. All e.s.d.'s (except the e.s.d. in the dihedral angle between two l.s. planes) are estimated using the full covariance matrix. The cell e.s.d.'s are taken into account individually in the estimation of e.s.d.'s in distances, angles and torsion angles; correlations between e.s.d.'s in cell parameters are only used when they are defined by crystal symmetry. An approximate (isotropic) treatment of cell e.s.d.'s is used for estimating e.s.d.'s involving l.s. planes.
Refinement. Refinement of *F*^2^ against ALL reflections. The weighted *R*-factor *wR* and goodness of fit *S* are based on *F*^2^, conventional *R*-factors *R* are based on *F*, with *F* set to zero for negative *F*^2^. The threshold expression of *F*^2^ > σ(*F*^2^) is used only for calculating *R*-factors(gt) *etc*. and is not relevant to the choice of reflections for refinement. *R*-factors based on *F*^2^ are statistically about twice as large as those based on *F*, and *R*- factors based on ALL data will be even larger.

### Fractional atomic coordinates and isotropic or equivalent isotropic displacement parameters (Å^2^)

**Table:**

	*x*	*y*	*z*	*U*_iso_*/*U*_eq_	
C1	0.8831 (4)	0.5306 (3)	0.1041 (4)	0.0406 (14)	
H1	0.8907	0.5862	0.0547	0.049*	
C2	0.8911 (4)	0.4450 (4)	0.0941 (4)	0.0564 (18)	
H2	0.9063	0.4293	0.0376	0.068*	
C3	0.8569 (3)	0.4366 (3)	0.2397 (4)	0.0408 (14)	
H3	0.8431	0.4115	0.3050	0.049*	
C4	0.6680 (4)	0.7430 (4)	0.3389 (4)	0.0506 (17)	
H4	0.6936	0.7779	0.3601	0.061*	
C5	0.5748 (4)	0.7497 (4)	0.3535 (4)	0.0613 (19)	
H5	0.5238	0.7872	0.3870	0.074*	
C6	0.6578 (4)	0.6494 (4)	0.2730 (4)	0.0485 (15)	
H6	0.6733	0.6043	0.2394	0.058*	
C7	0.8287 (4)	0.7768 (3)	0.0738 (4)	0.0514 (16)	
H7	0.7629	0.7785	0.0979	0.062*	
C8	0.8722 (5)	0.8207 (4)	−0.0143 (5)	0.069 (2)	
H8	0.8433	0.8580	−0.0624	0.083*	
C9	0.9751 (4)	0.7461 (3)	0.0637 (4)	0.0405 (14)	
H9	1.0337	0.7222	0.0786	0.049*	
C10	0.8820 (4)	0.7156 (3)	0.4120 (4)	0.0486 (16)	
H10	0.8740	0.6587	0.4596	0.058*	
C11	0.8913 (4)	0.7942 (4)	0.4237 (4)	0.0538 (17)	
H11	0.8904	0.8035	0.4798	0.065*	
C12	0.8984 (3)	0.8172 (3)	0.2799 (4)	0.0420 (15)	
H12	0.9041	0.8471	0.2160	0.050*	
C13	1.0812 (4)	0.5137 (4)	0.1793 (4)	0.0497 (17)	
H13	1.0588	0.4815	0.1522	0.060*	
C14	1.1718 (4)	0.5038 (4)	0.1747 (5)	0.066 (2)	
H14	1.2241	0.4649	0.1442	0.079*	
C15	1.0857 (4)	0.6036 (4)	0.2509 (4)	0.0482 (16)	
H15	1.0678	0.6492	0.2843	0.058*	
C16	0.9236 (4)	0.4531 (4)	0.4261 (4)	0.0562 (17)	
H16	0.9892	0.4450	0.3959	0.067*	
C17	0.8826 (5)	0.3985 (4)	0.5096 (5)	0.073 (2)	
H17	0.9134	0.3443	0.5471	0.088*	
C18	0.7769 (5)	0.5072 (3)	0.4562 (4)	0.0522 (17)	
H18	0.7165	0.5435	0.4516	0.063*	
C19	0.3554 (4)	0.0387 (4)	0.0896 (4)	0.0521 (17)	
H19	0.3463	0.0980	0.0455	0.063*	
C20	0.3532 (4)	−0.0454 (4)	0.0809 (4)	0.0610 (19)	
H20	0.3413	−0.0552	0.0307	0.073*	
C21	0.3814 (3)	−0.0680 (4)	0.2105 (4)	0.0495 (17)	
H21	0.3933	−0.0991	0.2692	0.059*	
C22	0.1618 (4)	0.2330 (4)	0.3056 (4)	0.0572 (18)	
H22	0.1804	0.2809	0.3164	0.069*	
C23	0.0712 (4)	0.2272 (5)	0.3179 (5)	0.079 (2)	
H23	0.0154	0.2689	0.3394	0.094*	
C24	0.1668 (4)	0.1106 (4)	0.2685 (4)	0.0498 (16)	
H24	0.1892	0.0549	0.2486	0.060*	
C25	0.2971 (4)	0.2983 (3)	0.0741 (4)	0.0421 (14)	
H25	0.2336	0.2989	0.1105	0.050*	
C26	0.3248 (4)	0.3585 (4)	−0.0112 (4)	0.0521 (16)	
H26	0.2861	0.4083	−0.0446	0.063*	
C27	0.4462 (4)	0.2595 (3)	0.0288 (4)	0.0431 (15)	
H27	0.5095	0.2279	0.0259	0.052*	
C28	0.3728 (4)	0.2350 (4)	0.3791 (5)	0.067 (2)	
H28	0.3601	0.1825	0.4311	0.081*	
C29	0.3862 (5)	0.3168 (4)	0.3815 (5)	0.076 (2)	
H29	0.3849	0.3321	0.4346	0.092*	
C30	0.3982 (4)	0.3240 (3)	0.2411 (4)	0.0489 (17)	
H30	0.4074	0.3474	0.1760	0.059*	
C31	0.5881 (4)	0.0190 (4)	0.1647 (4)	0.0497 (16)	
H31	0.5665	−0.0214	0.1461	0.060*	
C32	0.6772 (4)	0.0084 (4)	0.1642 (5)	0.073 (2)	
H32	0.7297	−0.0375	0.1445	0.088*	
C33	0.5884 (4)	0.1284 (4)	0.2165 (4)	0.0484 (16)	
H33	0.5696	0.1808	0.2413	0.058*	
C34	0.4485 (4)	−0.0236 (4)	0.4049 (4)	0.0511 (16)	
H34	0.5128	−0.0252	0.3715	0.061*	
C35	0.4172 (4)	−0.0696 (4)	0.4943 (4)	0.0550 (17)	
H35	0.4547	−0.1095	0.5345	0.066*	
C36	0.2995 (4)	0.0082 (4)	0.4388 (4)	0.0494 (16)	
H36	0.2366	0.0327	0.4346	0.059*	
C37	0.5915 (4)	0.4256 (3)	0.0643 (4)	0.0439 (15)	
H37	0.5389	0.4761	0.0614	0.053*	
C38	0.6337 (4)	0.3757 (4)	0.0021 (4)	0.0449 (15)	
H38	0.6162	0.3853	−0.0524	0.054*	
C39	0.7043 (4)	0.3218 (4)	0.1094 (4)	0.0529 (16)	
H39	0.7474	0.2842	0.1446	0.063*	
C40	0.5647 (4)	0.4377 (4)	0.3840 (4)	0.0502 (16)	
H40	0.5211	0.4956	0.3745	0.060*	
C41	0.5914 (4)	0.3905 (4)	0.4620 (4)	0.0500 (16)	
H41	0.5674	0.4089	0.5178	0.060*	
C42	0.6655 (4)	0.3135 (4)	0.3643 (4)	0.0457 (15)	
H42	0.7057	0.2660	0.3359	0.055*	
C43	0.8919 (4)	0.0898 (3)	−0.0391 (4)	0.0453 (15)	
H43	0.9103	0.0628	0.0168	0.054*	
C44	0.9266 (4)	0.0552 (3)	−0.1126 (4)	0.0410 (14)	
H44	0.9729	−0.0001	−0.1180	0.049*	
C45	0.8238 (4)	0.1831 (4)	−0.1424 (4)	0.0462 (16)	
H45	0.7855	0.2346	−0.1747	0.055*	
C46	0.9204 (4)	0.0970 (3)	0.5650 (4)	0.0405 (14)	
H46	0.9791	0.0543	0.5581	0.049*	
C47	0.8726 (4)	0.1345 (4)	0.5007 (4)	0.0469 (15)	
H47	0.8898	0.1239	0.4415	0.056*	
C48	0.7976 (4)	0.1849 (3)	0.6218 (4)	0.0427 (15)	
H48	0.7503	0.2179	0.6624	0.051*	
N1	0.8625 (3)	0.5252 (2)	0.1959 (3)	0.0332 (11)	
N2	0.8731 (3)	0.3863 (3)	0.1803 (3)	0.0449 (13)	
H49	0.8722	0.3261	0.1950	0.054*	
N3	0.7205 (3)	0.6795 (3)	0.2899 (3)	0.0378 (11)	
N4	0.5702 (3)	0.6900 (3)	0.3088 (3)	0.0463 (12)	
H50	0.5185	0.6803	0.3047	0.056*	
N5	0.8931 (3)	0.7305 (2)	0.1219 (3)	0.0352 (11)	
N6	0.9645 (4)	0.8004 (3)	−0.0195 (3)	0.0593 (15)	
H51	1.0099	0.8194	−0.0687	0.071*	
N7	0.8855 (3)	0.7292 (2)	0.3222 (3)	0.0322 (11)	
N8	0.9024 (3)	0.8574 (3)	0.3397 (3)	0.0417 (12)	
H52	0.9107	0.9151	0.3267	0.050*	
N9	1.0254 (3)	0.5760 (3)	0.2280 (3)	0.0394 (11)	
N10	1.1739 (3)	0.5608 (3)	0.2223 (4)	0.0571 (14)	
H53	1.2240	0.5678	0.2321	0.068*	
N11	0.8552 (3)	0.5217 (3)	0.3926 (3)	0.0360 (11)	
N12	0.7920 (4)	0.4350 (3)	0.5287 (4)	0.0783 (19)	
H54	0.7491	0.4153	0.5800	0.094*	
N13	0.3731 (3)	0.0232 (3)	0.1728 (3)	0.0383 (12)	
N14	0.3711 (3)	−0.1118 (3)	0.1568 (3)	0.0463 (13)	
H55	0.3753	−0.1727	0.1687	0.056*	
N15	0.2210 (3)	0.1589 (3)	0.2754 (3)	0.0389 (11)	
N16	0.0771 (4)	0.1505 (4)	0.2931 (4)	0.0773 (18)	
H56	0.0293	0.1301	0.2933	0.093*	
N17	0.3736 (3)	0.2371 (3)	0.0996 (3)	0.0355 (11)	
N18	0.4197 (3)	0.3317 (3)	−0.0379 (3)	0.0476 (12)	
H57	0.4574	0.3577	−0.0906	0.057*	
N19	0.3804 (3)	0.2397 (3)	0.2897 (3)	0.0443 (13)	
N20	0.4017 (4)	0.3723 (3)	0.2939 (4)	0.0691 (18)	
H58	0.4122	0.4299	0.2751	0.083*	
N21	0.5318 (3)	0.0937 (3)	0.1948 (3)	0.0361 (11)	
N22	0.6758 (3)	0.0780 (3)	0.1980 (4)	0.0661 (16)	
H59	0.7249	0.0886	0.2065	0.079*	
N23	0.3744 (3)	0.0256 (3)	0.3694 (3)	0.0376 (11)	
N24	0.3226 (3)	−0.0481 (3)	0.5157 (3)	0.0526 (13)	
H60	0.2835	−0.0675	0.5701	0.063*	
N25	0.6377 (3)	0.3910 (3)	0.1336 (3)	0.0469 (13)	
N26	0.7052 (3)	0.3096 (3)	0.0299 (3)	0.0536 (14)	
H61	0.7441	0.2676	0.0018	0.064*	
N27	0.6113 (3)	0.3877 (3)	0.3215 (3)	0.0506 (13)	
H62	0.6067	0.4013	0.2648	0.061*	
N28	0.6587 (3)	0.3115 (3)	0.4493 (3)	0.0561 (14)	
N29	0.8255 (3)	0.1704 (3)	−0.0570 (3)	0.0486 (13)	
N30	0.8833 (3)	0.1136 (3)	−0.1778 (3)	0.0452 (12)	
H63	0.8922	0.1074	−0.2330	0.054*	
N31	0.7945 (3)	0.1907 (3)	0.5383 (3)	0.0460 (12)	
H64	0.7496	0.2252	0.5119	0.055*	
N32	0.8733 (3)	0.1284 (3)	0.6417 (3)	0.0452 (12)	
O1	0.9397 (2)	0.0386 (2)	0.2964 (3)	0.0546 (11)	
O2	0.7783 (3)	0.1092 (3)	0.3067 (3)	0.0821 (14)	
O3	0.8813 (3)	0.1121 (4)	0.1626 (3)	0.1085 (18)	
O4	0.8938 (3)	0.1995 (2)	0.2527 (4)	0.113 (2)	
O5	0.4011 (3)	0.6542 (2)	0.3180 (3)	0.0648 (13)	
O6	0.4728 (3)	0.5586 (2)	0.2131 (3)	0.0780 (14)	
O7	0.3098 (3)	0.5833 (3)	0.2806 (3)	0.0946 (17)	
O8	0.3793 (3)	0.7069 (2)	0.1671 (3)	0.0575 (12)	
S1	0.87346 (9)	0.11509 (8)	0.25340 (9)	0.0254 (3)	
S2	0.39014 (9)	0.62584 (8)	0.24501 (9)	0.0286 (3)	
Fe1	0.87273 (5)	0.62872 (4)	0.25814 (5)	0.03015 (19)	
Fe2	0.37628 (5)	0.12942 (5)	0.23314 (6)	0.0351 (2)	

### Atomic displacement parameters (Å^2^)

**Table:**

	*U*^11^	*U*^22^	*U*^33^	*U*^12^	*U*^13^	*U*^23^
C1	0.057 (4)	0.037 (3)	0.027 (3)	−0.016 (3)	−0.011 (3)	−0.003 (3)
C2	0.079 (5)	0.054 (4)	0.040 (4)	−0.018 (3)	−0.013 (4)	−0.016 (3)
C3	0.051 (4)	0.040 (3)	0.036 (4)	−0.015 (3)	−0.014 (3)	−0.009 (3)
C4	0.042 (4)	0.048 (4)	0.064 (5)	−0.009 (3)	−0.017 (4)	−0.015 (3)
C5	0.050 (4)	0.062 (4)	0.075 (6)	0.003 (3)	−0.014 (4)	−0.036 (4)
C6	0.049 (4)	0.061 (4)	0.034 (4)	−0.009 (3)	−0.012 (3)	−0.012 (3)
C7	0.056 (4)	0.052 (4)	0.037 (4)	−0.020 (3)	−0.019 (4)	0.012 (3)
C8	0.074 (5)	0.077 (5)	0.051 (5)	−0.015 (4)	−0.038 (4)	0.012 (4)
C9	0.046 (4)	0.033 (3)	0.033 (4)	−0.013 (3)	−0.001 (3)	−0.004 (3)
C10	0.087 (5)	0.034 (3)	0.030 (4)	−0.023 (3)	−0.014 (3)	−0.008 (3)
C11	0.078 (5)	0.049 (4)	0.039 (4)	−0.016 (3)	−0.010 (4)	−0.021 (3)
C12	0.057 (4)	0.032 (3)	0.043 (4)	−0.017 (3)	−0.015 (3)	−0.009 (3)
C13	0.036 (4)	0.053 (4)	0.071 (5)	0.000 (3)	−0.015 (3)	−0.037 (4)
C14	0.050 (4)	0.061 (4)	0.084 (6)	0.009 (4)	−0.023 (4)	−0.028 (4)
C15	0.040 (4)	0.055 (4)	0.059 (5)	−0.008 (3)	−0.020 (3)	−0.020 (3)
C16	0.055 (4)	0.062 (4)	0.049 (5)	−0.019 (4)	−0.019 (4)	−0.001 (3)
C17	0.103 (6)	0.057 (4)	0.048 (5)	−0.013 (5)	−0.023 (5)	0.002 (4)
C18	0.079 (5)	0.034 (3)	0.029 (4)	−0.009 (3)	−0.007 (4)	0.000 (3)
C19	0.091 (5)	0.039 (3)	0.027 (4)	−0.021 (3)	−0.018 (4)	0.000 (3)
C20	0.101 (6)	0.050 (4)	0.042 (4)	−0.032 (4)	−0.009 (4)	−0.020 (3)
C21	0.049 (4)	0.043 (3)	0.071 (5)	−0.006 (3)	−0.021 (4)	−0.030 (4)
C22	0.045 (4)	0.043 (4)	0.067 (5)	0.000 (3)	−0.005 (4)	−0.013 (3)
C23	0.029 (4)	0.096 (6)	0.066 (6)	0.018 (4)	0.001 (4)	−0.007 (5)
C24	0.041 (4)	0.084 (4)	0.029 (4)	−0.032 (4)	−0.008 (3)	−0.005 (3)
C25	0.035 (3)	0.053 (3)	0.028 (3)	−0.018 (3)	−0.004 (3)	0.004 (3)
C26	0.033 (4)	0.064 (4)	0.045 (4)	−0.007 (3)	−0.003 (3)	−0.007 (3)
C27	0.029 (3)	0.048 (4)	0.059 (5)	−0.010 (3)	−0.007 (3)	−0.025 (3)
C28	0.084 (5)	0.078 (5)	0.048 (5)	−0.039 (4)	0.003 (4)	−0.028 (4)
C29	0.115 (7)	0.067 (5)	0.072 (6)	−0.020 (4)	−0.036 (5)	−0.037 (4)
C30	0.058 (4)	0.040 (3)	0.063 (5)	−0.013 (3)	−0.023 (4)	−0.022 (3)
C31	0.035 (4)	0.060 (4)	0.055 (5)	−0.011 (3)	−0.003 (3)	−0.025 (3)
C32	0.038 (4)	0.091 (5)	0.092 (7)	−0.004 (4)	−0.017 (4)	−0.034 (5)
C33	0.045 (4)	0.061 (4)	0.052 (4)	−0.016 (3)	−0.027 (3)	−0.012 (3)
C34	0.045 (4)	0.061 (4)	0.041 (4)	−0.011 (3)	−0.012 (3)	−0.005 (3)
C35	0.041 (4)	0.069 (4)	0.048 (5)	−0.006 (3)	−0.014 (4)	−0.011 (4)
C36	0.047 (4)	0.063 (4)	0.031 (4)	−0.026 (3)	−0.003 (3)	0.001 (3)
C37	0.052 (4)	0.041 (3)	0.044 (4)	−0.010 (3)	−0.019 (3)	−0.010 (3)
C38	0.051 (4)	0.065 (4)	0.021 (3)	−0.019 (3)	−0.012 (3)	−0.007 (3)
C39	0.073 (5)	0.063 (4)	0.024 (4)	−0.020 (4)	−0.013 (3)	−0.008 (3)
C40	0.062 (4)	0.050 (4)	0.040 (4)	−0.010 (3)	−0.012 (4)	−0.016 (3)
C41	0.063 (4)	0.046 (4)	0.032 (4)	−0.006 (3)	−0.006 (3)	−0.010 (3)
C42	0.045 (4)	0.038 (3)	0.052 (5)	0.005 (3)	−0.023 (3)	−0.012 (3)
C43	0.066 (4)	0.049 (4)	0.029 (4)	−0.018 (3)	−0.017 (3)	−0.011 (3)
C44	0.044 (4)	0.030 (3)	0.042 (4)	0.001 (3)	−0.014 (3)	−0.004 (3)
C45	0.046 (4)	0.037 (3)	0.059 (5)	0.004 (3)	−0.023 (3)	−0.018 (3)
C46	0.029 (3)	0.047 (3)	0.047 (4)	0.003 (3)	−0.013 (3)	−0.021 (3)
C47	0.045 (4)	0.081 (4)	0.023 (4)	−0.016 (3)	−0.005 (3)	−0.025 (3)
C48	0.047 (4)	0.039 (3)	0.038 (4)	−0.001 (3)	−0.010 (3)	−0.014 (3)
N1	0.038 (3)	0.025 (2)	0.036 (3)	−0.007 (2)	−0.014 (2)	−0.003 (2)
N2	0.057 (3)	0.027 (2)	0.058 (4)	−0.005 (2)	−0.023 (3)	−0.016 (3)
N3	0.040 (3)	0.046 (3)	0.025 (3)	−0.014 (2)	−0.006 (2)	−0.005 (2)
N4	0.035 (3)	0.063 (3)	0.042 (3)	−0.013 (3)	−0.014 (3)	−0.009 (3)
N5	0.047 (3)	0.024 (2)	0.031 (3)	−0.010 (2)	−0.010 (2)	0.000 (2)
N6	0.088 (4)	0.045 (3)	0.031 (3)	−0.022 (3)	−0.003 (3)	0.001 (3)
N7	0.036 (3)	0.031 (2)	0.034 (3)	−0.015 (2)	−0.007 (2)	−0.009 (2)
N8	0.060 (3)	0.024 (2)	0.047 (3)	−0.011 (2)	−0.017 (3)	−0.012 (2)
N9	0.040 (3)	0.043 (3)	0.034 (3)	−0.015 (2)	−0.009 (2)	−0.003 (2)
N10	0.045 (3)	0.060 (3)	0.070 (4)	−0.013 (3)	−0.020 (3)	−0.017 (3)
N11	0.053 (3)	0.029 (2)	0.031 (3)	−0.017 (2)	−0.011 (3)	−0.006 (2)
N12	0.119 (5)	0.054 (3)	0.022 (3)	−0.010 (4)	0.017 (4)	−0.004 (3)
N13	0.040 (3)	0.029 (2)	0.050 (3)	−0.013 (2)	−0.013 (3)	−0.010 (2)
N14	0.057 (3)	0.031 (3)	0.055 (4)	−0.015 (2)	−0.010 (3)	−0.018 (3)
N15	0.035 (3)	0.033 (2)	0.040 (3)	−0.015 (2)	−0.012 (2)	0.010 (2)
N16	0.037 (4)	0.137 (5)	0.052 (4)	−0.022 (4)	−0.020 (3)	−0.007 (4)
N17	0.036 (3)	0.034 (2)	0.038 (3)	−0.015 (2)	−0.010 (2)	−0.005 (2)
N18	0.060 (4)	0.057 (3)	0.024 (3)	−0.023 (3)	−0.004 (3)	−0.007 (2)
N19	0.048 (3)	0.044 (3)	0.048 (4)	−0.022 (2)	−0.002 (3)	−0.021 (3)
N20	0.099 (5)	0.033 (3)	0.097 (5)	0.001 (3)	−0.055 (4)	−0.028 (3)
N21	0.028 (3)	0.052 (3)	0.029 (3)	−0.014 (2)	−0.006 (2)	−0.009 (2)
N22	0.046 (4)	0.073 (4)	0.088 (5)	−0.016 (3)	−0.044 (3)	−0.003 (3)
N23	0.042 (3)	0.037 (3)	0.035 (3)	−0.011 (2)	−0.010 (2)	−0.010 (2)
N24	0.048 (3)	0.063 (3)	0.040 (4)	−0.012 (3)	−0.006 (3)	−0.012 (3)
N25	0.052 (3)	0.048 (3)	0.049 (4)	−0.002 (3)	−0.017 (3)	−0.026 (3)
N26	0.049 (3)	0.066 (3)	0.048 (4)	−0.006 (3)	−0.005 (3)	−0.031 (3)
N27	0.054 (3)	0.061 (3)	0.046 (4)	−0.009 (3)	−0.020 (3)	−0.021 (3)
N28	0.067 (4)	0.046 (3)	0.041 (4)	0.004 (3)	−0.016 (3)	−0.006 (3)
N29	0.044 (3)	0.058 (3)	0.049 (4)	0.002 (3)	−0.017 (3)	−0.027 (3)
N30	0.047 (3)	0.047 (3)	0.049 (4)	−0.011 (2)	−0.016 (3)	−0.017 (3)
N31	0.055 (3)	0.051 (3)	0.036 (3)	−0.016 (3)	−0.019 (3)	−0.006 (2)
N32	0.055 (3)	0.044 (3)	0.039 (3)	−0.008 (3)	−0.018 (3)	−0.010 (2)
O1	0.064 (3)	0.038 (2)	0.070 (3)	0.0048 (19)	−0.043 (3)	−0.012 (2)
O2	0.040 (3)	0.154 (4)	0.042 (3)	−0.025 (3)	0.001 (2)	−0.022 (3)
O3	0.079 (4)	0.205 (5)	0.046 (3)	0.028 (3)	−0.031 (3)	−0.071 (4)
O4	0.103 (4)	0.019 (2)	0.229 (7)	−0.003 (2)	−0.091 (4)	−0.009 (3)
O5	0.063 (3)	0.089 (3)	0.070 (3)	0.007 (2)	−0.042 (3)	−0.049 (3)
O6	0.079 (3)	0.059 (2)	0.051 (3)	0.025 (2)	0.002 (3)	−0.013 (2)
O7	0.092 (4)	0.138 (4)	0.071 (4)	−0.092 (3)	−0.017 (3)	0.003 (3)
O8	0.092 (3)	0.033 (2)	0.038 (3)	0.001 (2)	−0.022 (3)	−0.0052 (19)
S1	0.0245 (7)	0.0285 (7)	0.0213 (7)	−0.0020 (6)	−0.0054 (6)	−0.0080 (6)
S2	0.0324 (8)	0.0315 (7)	0.0235 (8)	−0.0044 (6)	−0.0084 (6)	−0.0099 (6)
Fe1	0.0341 (5)	0.0300 (4)	0.0259 (5)	−0.0140 (3)	−0.0048 (4)	−0.0040 (3)
Fe2	0.0347 (5)	0.0354 (4)	0.0384 (5)	−0.0168 (4)	−0.0051 (4)	−0.0107 (4)

### Geometric parameters (Å, °)

**Table:**

C1—C2	1.351 (6)	C31—H31	0.9500
C1—N1	1.369 (6)	C32—N22	1.353 (6)
C1—H1	0.9500	C32—H32	0.9500
C2—N2	1.349 (6)	C33—N21	1.332 (5)
C2—H2	0.9500	C33—N22	1.337 (6)
C3—N1	1.318 (5)	C33—H33	0.9500
C3—N2	1.342 (6)	C34—C35	1.342 (7)
C3—H3	0.9500	C34—N23	1.360 (6)
C4—C5	1.353 (7)	C34—H34	0.9500
C4—N3	1.366 (6)	C35—N24	1.345 (6)
C4—H4	0.9500	C35—H35	0.9500
C5—N4	1.375 (6)	C36—N23	1.315 (6)
C5—H5	0.9500	C36—N24	1.334 (7)
C6—N3	1.323 (6)	C36—H36	0.9500
C6—N4	1.330 (6)	C37—C38	1.342 (6)
C6—H6	0.9500	C37—N25	1.391 (6)
C7—N5	1.349 (6)	C37—H37	0.9500
C7—C8	1.360 (8)	C38—N26	1.352 (6)
C7—H7	0.9500	C38—H38	0.9500
C8—N6	1.344 (7)	C39—N25	1.313 (6)
C8—H8	0.9500	C39—N26	1.343 (6)
C9—N5	1.318 (6)	C39—H39	0.9500
C9—N6	1.341 (6)	C40—C41	1.344 (7)
C9—H9	0.9500	C40—N27	1.352 (6)
C10—C11	1.345 (6)	C40—H40	0.9500
C10—N7	1.362 (6)	C41—N28	1.379 (6)
C10—H10	0.9500	C41—H41	0.9500
C11—N8	1.349 (6)	C42—N28	1.316 (7)
C11—H11	0.9500	C42—N27	1.334 (6)
C12—N7	1.331 (5)	C42—H42	0.9500
C12—N8	1.333 (6)	C43—C44	1.338 (6)
C12—H12	0.9500	C43—N29	1.379 (6)
C13—C14	1.339 (7)	C43—H43	0.9500
C13—N9	1.361 (6)	C44—N30	1.352 (6)
C13—H13	0.9500	C44—H44	0.9500
C14—N10	1.361 (6)	C45—N29	1.319 (7)
C14—H14	0.9500	C45—N30	1.344 (6)
C15—N9	1.324 (5)	C45—H45	0.9500
C15—N10	1.325 (6)	C46—C47	1.341 (7)
C15—H15	0.9500	C46—N32	1.365 (6)
C16—C17	1.359 (8)	C46—H46	0.9500
C16—N11	1.368 (6)	C47—N31	1.350 (6)
C16—H16	0.9500	C47—H47	0.9500
C17—N12	1.317 (7)	C48—N32	1.303 (6)
C17—H17	0.9500	C48—N31	1.323 (6)
C18—N11	1.302 (6)	C48—H48	0.9500
C18—N12	1.336 (7)	N1—Fe1	2.218 (4)
C18—H18	0.9500	N2—H49	0.8800
C19—C20	1.363 (6)	N3—Fe1	2.185 (4)
C19—N13	1.377 (6)	N4—H50	0.8800
C19—H19	0.9500	N5—Fe1	2.184 (4)
C20—N14	1.344 (7)	N6—H51	0.8800
C20—H20	0.9500	N7—Fe1	2.215 (3)
C21—N13	1.314 (6)	N8—H52	0.8800
C21—N14	1.332 (6)	N9—Fe1	2.195 (4)
C21—H21	0.9500	N10—H53	0.8800
C22—N15	1.361 (6)	N11—Fe1	2.199 (4)
C22—C23	1.367 (7)	N12—H54	0.8800
C22—H22	0.9500	N13—Fe2	2.189 (4)
C23—N16	1.344 (7)	N14—H55	0.8800
C23—H23	0.9500	N15—Fe2	2.205 (4)
C24—N15	1.321 (5)	N16—H56	0.8800
C24—N16	1.322 (6)	N17—Fe2	2.213 (4)
C24—H24	0.9500	N18—H57	0.8800
C25—C26	1.364 (7)	N19—Fe2	2.211 (4)
C25—N17	1.368 (6)	N20—H58	0.8800
C25—H25	0.9500	N21—Fe2	2.210 (4)
C26—N18	1.355 (6)	N22—H59	0.8800
C26—H26	0.9500	N23—Fe2	2.211 (4)
C27—N17	1.318 (6)	N24—H60	0.8800
C27—N18	1.328 (6)	N26—H61	0.8800
C27—H27	0.9500	N27—H62	0.8800
C28—C29	1.353 (6)	N30—H63	0.8800
C28—N19	1.370 (7)	N31—H64	0.8800
C28—H28	0.9500	O1—S1	1.460 (3)
C29—N20	1.347 (7)	O2—S1	1.433 (4)
C29—H29	0.9500	O3—S1	1.431 (4)
C30—N19	1.318 (6)	O4—S1	1.422 (3)
C30—N20	1.328 (6)	O5—S2	1.454 (3)
C30—H30	0.9500	O6—S2	1.454 (4)
C31—C32	1.332 (7)	O7—S2	1.421 (3)
C31—N21	1.355 (5)	O8—S2	1.446 (4)
			
C2—C1—N1	110.0 (5)	N32—C46—H46	124.6
C2—C1—H1	125.0	C46—C47—N31	105.0 (5)
N1—C1—H1	125.0	C46—C47—H47	127.5
N2—C2—C1	106.3 (5)	N31—C47—H47	127.5
N2—C2—H2	126.9	N32—C48—N31	111.9 (5)
C1—C2—H2	126.9	N32—C48—H48	124.1
N1—C3—N2	111.4 (5)	N31—C48—H48	124.1
N1—C3—H3	124.3	C3—N1—C1	104.8 (4)
N2—C3—H3	124.3	C3—N1—Fe1	125.1 (4)
C5—C4—N3	111.3 (5)	C1—N1—Fe1	128.3 (3)
C5—C4—H4	124.4	C3—N2—C2	107.4 (4)
N3—C4—H4	124.4	C3—N2—H49	126.3
C4—C5—N4	104.5 (5)	C2—N2—H49	126.3
C4—C5—H5	127.7	C6—N3—C4	104.6 (4)
N4—C5—H5	127.7	C6—N3—Fe1	127.4 (4)
N3—C6—N4	111.6 (5)	C4—N3—Fe1	127.8 (3)
N3—C6—H6	124.2	C6—N4—C5	108.1 (5)
N4—C6—H6	124.2	C6—N4—H50	126.0
N5—C7—C8	109.8 (5)	C5—N4—H50	126.0
N5—C7—H7	125.1	C9—N5—C7	105.8 (5)
C8—C7—H7	125.1	C9—N5—Fe1	125.4 (4)
N6—C8—C7	106.0 (6)	C7—N5—Fe1	127.8 (4)
N6—C8—H8	127.0	C9—N6—C8	107.7 (5)
C7—C8—H8	127.0	C9—N6—H51	126.1
N5—C9—N6	110.7 (5)	C8—N6—H51	126.1
N5—C9—H9	124.7	C12—N7—C10	105.0 (4)
N6—C9—H9	124.7	C12—N7—Fe1	126.7 (4)
C11—C10—N7	110.3 (5)	C10—N7—Fe1	128.3 (3)
C11—C10—H10	124.9	C12—N8—C11	108.2 (4)
N7—C10—H10	124.9	C12—N8—H52	125.9
C10—C11—N8	106.1 (5)	C11—N8—H52	125.9
C10—C11—H11	127.0	C15—N9—C13	103.7 (4)
N8—C11—H11	127.0	C15—N9—Fe1	125.9 (4)
N7—C12—N8	110.6 (5)	C13—N9—Fe1	130.4 (3)
N7—C12—H12	124.7	C15—N10—C14	106.4 (5)
N8—C12—H12	124.7	C15—N10—H53	126.8
C14—C13—N9	110.8 (5)	C14—N10—H53	126.8
C14—C13—H13	124.6	C18—N11—C16	104.8 (5)
N9—C13—H13	124.6	C18—N11—Fe1	127.6 (4)
C13—C14—N10	106.3 (5)	C16—N11—Fe1	127.5 (4)
C13—C14—H14	126.9	C17—N12—C18	107.7 (6)
N10—C14—H14	126.8	C17—N12—H54	126.1
N9—C15—N10	112.7 (5)	C18—N12—H54	126.1
N9—C15—H15	123.7	C21—N13—C19	105.0 (4)
N10—C15—H15	123.7	C21—N13—Fe2	127.7 (4)
C17—C16—N11	108.9 (6)	C19—N13—Fe2	127.1 (3)
C17—C16—H16	125.6	C21—N14—C20	107.0 (4)
N11—C16—H16	125.6	C21—N14—H55	126.5
N12—C17—C16	106.8 (6)	C20—N14—H55	126.5
N12—C17—H17	126.6	C24—N15—C22	106.0 (4)
C16—C17—H17	126.6	C24—N15—Fe2	125.6 (4)
N11—C18—N12	111.7 (6)	C22—N15—Fe2	128.2 (3)
N11—C18—H18	124.2	C24—N16—C23	108.7 (5)
N12—C18—H18	124.2	C24—N16—H56	125.7
C20—C19—N13	108.6 (5)	C23—N16—H56	125.7
C20—C19—H19	125.7	C27—N17—C25	104.8 (5)
N13—C19—H19	125.7	C27—N17—Fe2	127.4 (4)
N14—C20—C19	107.1 (5)	C25—N17—Fe2	127.8 (4)
N14—C20—H20	126.5	C27—N18—C26	108.5 (5)
C19—C20—H20	126.5	C27—N18—H57	125.8
N13—C21—N14	112.2 (5)	C26—N18—H57	125.8
N13—C21—H21	123.9	C30—N19—C28	104.8 (4)
N14—C21—H21	123.9	C30—N19—Fe2	125.8 (4)
N15—C22—C23	108.8 (5)	C28—N19—Fe2	129.2 (4)
N15—C22—H22	125.6	C30—N20—C29	107.3 (5)
C23—C22—H22	125.6	C30—N20—H58	126.3
N16—C23—C22	105.9 (6)	C29—N20—H58	126.3
N16—C23—H23	127.0	C33—N21—C31	104.5 (4)
C22—C23—H23	127.0	C33—N21—Fe2	126.1 (4)
N15—C24—N16	110.7 (5)	C31—N21—Fe2	127.8 (3)
N15—C24—H24	124.7	C33—N22—C32	109.6 (5)
N16—C24—H24	124.7	C33—N22—H59	125.2
C26—C25—N17	110.3 (5)	C32—N22—H59	125.2
C26—C25—H25	124.9	C36—N23—C34	104.6 (5)
N17—C25—H25	124.9	C36—N23—Fe2	126.5 (4)
N18—C26—C25	104.9 (5)	C34—N23—Fe2	128.3 (4)
N18—C26—H26	127.5	C36—N24—C35	106.9 (5)
C25—C26—H26	127.5	C36—N24—H60	126.6
N17—C27—N18	111.5 (5)	C35—N24—H60	126.6
N17—C27—H27	124.2	C39—N25—C37	104.4 (5)
N18—C27—H27	124.2	C39—N26—C38	105.3 (5)
C29—C28—N19	109.3 (6)	C39—N26—H61	127.3
C29—C28—H28	125.3	C38—N26—H61	127.3
N19—C28—H28	125.3	C42—N27—C40	105.2 (5)
N20—C29—C28	106.6 (6)	C42—N27—H62	127.4
N20—C29—H29	126.7	C40—N27—H62	127.4
C28—C29—H29	126.7	C42—N28—C41	102.7 (5)
N19—C30—N20	111.9 (6)	C45—N29—C43	104.9 (4)
N19—C30—H30	124.0	C45—N30—C44	107.0 (5)
N20—C30—H30	124.0	C45—N30—H63	126.5
C32—C31—N21	112.4 (5)	C44—N30—H63	126.5
C32—C31—H31	123.8	C48—N31—C47	108.0 (5)
N21—C31—H31	123.8	C48—N31—H64	126.0
C31—C32—N22	104.1 (5)	C47—N31—H64	126.0
C31—C32—H32	128.0	C48—N32—C46	104.3 (5)
N22—C32—H32	128.0	O4—S1—O3	112.3 (3)
N21—C33—N22	109.4 (5)	O4—S1—O2	109.2 (3)
N21—C33—H33	125.3	O3—S1—O2	106.9 (3)
N22—C33—H33	125.3	O4—S1—O1	105.9 (2)
C35—C34—N23	110.0 (5)	O3—S1—O1	111.7 (2)
C35—C34—H34	125.0	O2—S1—O1	110.9 (2)
N23—C34—H34	125.0	O7—S2—O8	110.4 (3)
C34—C35—N24	106.6 (6)	O7—S2—O5	108.7 (3)
C34—C35—H35	126.7	O8—S2—O5	109.9 (2)
N24—C35—H35	126.7	O7—S2—O6	109.2 (3)
N23—C36—N24	111.8 (5)	O8—S2—O6	108.1 (2)
N23—C36—H36	124.1	O5—S2—O6	110.5 (2)
N24—C36—H36	124.1	N5—Fe1—N3	91.34 (16)
C38—C37—N25	108.5 (5)	N5—Fe1—N9	89.46 (16)
C38—C37—H37	125.7	N3—Fe1—N9	179.19 (16)
N25—C37—H37	125.7	N5—Fe1—N11	177.69 (15)
C37—C38—N26	108.6 (5)	N3—Fe1—N11	89.94 (16)
C37—C38—H38	125.7	N9—Fe1—N11	89.25 (16)
N26—C38—H38	125.7	N5—Fe1—N7	91.76 (15)
N25—C39—N26	113.1 (5)	N3—Fe1—N7	89.64 (15)
N25—C39—H39	123.4	N9—Fe1—N7	90.47 (14)
N26—C39—H39	123.4	N11—Fe1—N7	90.17 (14)
C41—C40—N27	107.5 (5)	N5—Fe1—N1	89.24 (15)
C41—C40—H40	126.3	N3—Fe1—N1	91.41 (15)
N27—C40—H40	126.3	N9—Fe1—N1	88.46 (15)
C40—C41—N28	110.2 (5)	N11—Fe1—N1	88.80 (14)
C40—C41—H41	124.9	N7—Fe1—N1	178.53 (15)
N28—C41—H41	124.9	N13—Fe2—N15	88.93 (15)
N28—C42—N27	114.4 (5)	N13—Fe2—N21	89.99 (15)
N28—C42—H42	122.8	N15—Fe2—N21	177.53 (15)
N27—C42—H42	122.8	N13—Fe2—N19	178.24 (18)
C44—C43—N29	109.6 (5)	N15—Fe2—N19	91.41 (16)
C44—C43—H43	125.2	N21—Fe2—N19	89.74 (15)
N29—C43—H43	125.2	N13—Fe2—N23	91.88 (15)
C43—C44—N30	107.2 (5)	N15—Fe2—N23	88.94 (15)
C43—C44—H44	126.4	N21—Fe2—N23	88.88 (16)
N30—C44—H44	126.4	N19—Fe2—N23	89.85 (16)
N29—C45—N30	111.3 (5)	N13—Fe2—N17	89.60 (15)
N29—C45—H45	124.3	N15—Fe2—N17	89.37 (16)
N30—C45—H45	124.3	N21—Fe2—N17	92.84 (15)
C47—C46—N32	110.8 (5)	N19—Fe2—N17	88.68 (16)
C47—C46—H46	124.6	N23—Fe2—N17	177.73 (16)

### Hydrogen-bond geometry (Å, °)

**Table:**

*D*—H···*A*	*D*—H	H···*A*	*D*···*A*	*D*—H···*A*
N2—H49···O4	0.88	1.84	2.679 (5)	160
N4—H50···O5	0.88	1.89	2.762 (5)	170
N6—H51···O3^i^	0.88	2.09	2.949 (7)	163
N8—H52···O1^ii^	0.88	1.94	2.822 (5)	176
N10—H53···O7^iii^	0.88	1.85	2.711 (6)	166
N12—H54···O7^iv^	0.88	2.10	2.867 (7)	145
N12—H54···O5^iv^	0.88	2.61	3.434 (7)	157
N14—H55···O8^v^	0.88	1.85	2.716 (5)	167
N16—H56···O1^vi^	0.88	2.22	3.074 (6)	163
N16—H56···O4^vi^	0.88	2.26	2.942 (6)	135
N18—H57···O6^vii^	0.88	2.07	2.937 (6)	169
N18—H57···O8^vii^	0.88	2.47	3.100 (6)	129
N20—H58···O6	0.88	2.21	3.071 (6)	166
N20—H58···O7	0.88	2.45	3.120 (6)	133
N22—H59···O2	0.88	2.18	2.939 (6)	144
N22—H59···O3	0.88	2.37	3.186 (6)	155
N24—H60···O2^viii^	0.88	1.84	2.710 (6)	168
N26—H61···N29	0.88	1.95	2.825 (6)	177
N27—H62···N25	0.88	2.04	2.875 (6)	158
N30—H63···N32^ix^	0.88	2.02	2.869 (6)	160
N31—H64···N28	0.88	1.92	2.800 (6)	174

Symmetry codes: (i) −*x*+2, −*y*+1, −*z*; (ii) *x*, *y*+1, *z*; (iii) *x*+1, *y*, *z*; (iv) −*x*+1, −*y*+1, −*z*+1; (v) *x*, *y*−1, *z*; (vi) *x*−1, *y*, *z*; (vii) −*x*+1, −*y*+1, −*z*; (viii) −*x*+1, −*y*, −*z*+1; (ix) *x*, *y*, *z*−1.
